# A methodology for projecting hospital bed need: a Michigan case study

**DOI:** 10.1186/1751-0473-5-4

**Published:** 2010-03-25

**Authors:** Shaun A Langley, Steven P Fuller, Joseph P Messina, Ashton M Shortridge, Sue C Grady

**Affiliations:** 1 Department of Geography, Michigan State University, East Lansing, MI, USA; 2 Center for Global Change and Earth Observations, Michigan State University, East Lansing, MI, USA; 3 Michigan Agricultural Experiment Station, Michigan State University, East Lansing, MI, USA

## Abstract

Michigan's Department of Community Health (MDCH) is responsible for managing hospitals through the utilization of a Certificate of Need (CON) Commission. Regulation is achieved by limiting the number of beds a hospital can use for inpatient services. MDCH assigns hospitals to service areas and sub areas by use patterns. Hospital beds are then assigned within these Hospital Service Areas and Facility Sub Areas. The determination of the number of hospital beds a facility subarea is authorized to hold, called bed need, is defined in the Michigan Hospital Standards and published by the CON Commission and MDCH. These standards vaguely define a methodology for calculating hospital bed need for a projection year, five years ahead of the base year (defined as the most recent year for which patient data have been published by the Michigan Hospital Association). MDCH approached the authors and requested a reformulation of the process. Here we present a comprehensive guide and associated code as interpreted from the hospital standards with results from the 2011 projection year. Additionally, we discuss methodologies for other states and compare them to Michigan's Bed Need methodology.

## Background

The Michigan Department of Community Health (MDCH) was founded in 1996 through the consolidation of the Department of Public Health, Department of Mental Health, and the state's Medicaid Agency. MDCH is tasked with preparing and administering many critical health services used by Michigan residents. The more well known services include Medicaid state health insurance, mental health services, health promotion and disease prevention programs.

**Figure 1:**
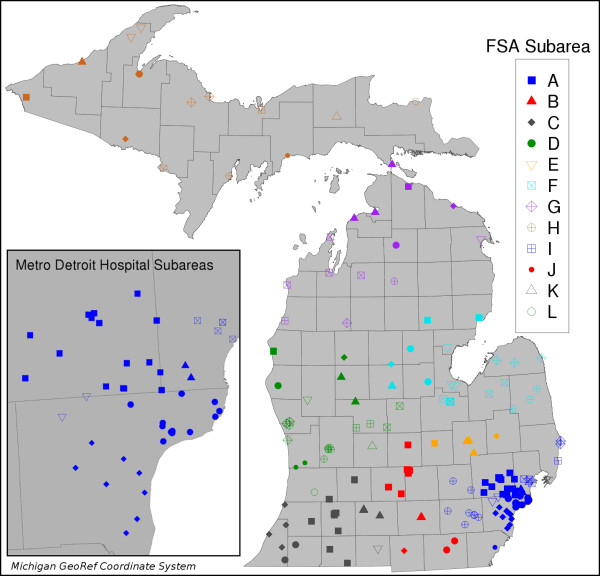
**Michigan Facility Service Areas**. Hues indicate Heath Service Areas (HSAs); symbols indicate Facility Subareas (FSA) within each HSA.

The Certificate of Need (CON) Commission is a state mandated program, operating under MDCH, tasked with balancing the cost, quality, and access of health care services within the state. The CON Commission regulates the number of inpatient beds each hospital is approved to operate. These beds are managed to meet, but not exceed, the demand for health care services in an area (i.e. "bed need"). Whenever there is a desire to open a new hospital facility, relocate hospital services, or expand existing facilities, a CON application is made to MDCH for the approval of additional beds. The MDCH evaluates these applications according to the anticipated "bed need" within the state for health care services. In Michigan, all regulatory decisions are made by aggregating zip codes and facilities into groups that reflect use patterns; these groupings reflect the geographic distribution of the state's population, use rates, and hospitals (Figure 1).

The original methodology for calculating bed need was adopted by the State-Wide Health Planning Commission in 1977 as a means for predicting future patient bed need [1]. The methodology achieves this objective by multiplying use rates in a base year and scaling them based on projected population growth. Between 1977 and 2008, the Michigan bed need assessment was completed in house by MDCH personnel.

In May 2009, prompted by questions over the interpretation of the bed need methodology, the authors were commissioned by MDCH to calculate the projected bed need for 2011 [2]. In the process of conducting the calculations and consulting with MDCH personnel, substantial uncertainty arose about implementing the methodology as described in the CON Hospital Bed Need standards. We clarified the standards by providing a detailed description of the methodology, including code, presented here used to calculate the bed need projections for the State of Michigan. This paper reports on the development of an unambiguous bed need methodology that fits the language of the Standard. This process is greatly facilitated by the use of standard query language (SQL) directly in the methodology report, in conjunction with natural language. Here we compare the approach with those used in other states and regions, and demonstrate important differences of spatial scale in the Michigan case.

## Methodology

The methodology utilized in this paper employs and rigorously interprets the Michigan CON Hospital Beds Review Standard (effective 3/17/2007), parts of which are summarized in this section. Our intent is to provide sufficient description of the methodology to enable interested parties to reproduce our results or implement the same methodology on other populations and data. We do not strive to justify the CON's rationale for calculating bed need in the manner specified; instead we aim to provide our interpretation of the methodology as it has been adopted. The calculation of bed need is facilitated through a set of relational database operations (in MySQL). SQL is an effective means for communicating methodology, as it leaves no room for misinterpretation of the steps. The ambiguity of the plain-English definition of the methodology, as laid out in the Hospital Beds Review Standard, was the impetus for this paper and the resulting methodology. The specific implementation of the code for the 2011 Michigan Bed Need is also available (see Additional File 1). We define the variables in general terms to facilitate the implementation of this code by other parties who may not utilize our specific data set.

In developing this code, we employ three data sets to calculate the Michigan 2011 Statewide bed need projections.

1) 2006 base year data from the Michigan Inpatient Database (MIDB)

2) Limited Access Area (LAA) zip codes (updated May 2008)

3) 2011 population projections by zip code (source: Claritas)

Inpatient data (MIDB) and population projections for Michigan (Claritas) are provided to the authors semi-annually. With these data, we calculated an updated LAA model in early 2008 (see Messina et al. [3] for a description of the methodology). Limited Access Areas are computed every two years in conjunction with the calculation of Bed Need, but do not affect the outcome of the distribution of beds to any region. Therefore, although mentioned here as a necessary data source and required by the current standards, we do not discuss further the calculation or utilization of LAA zip codes by MDCH.

### Definition of Variables

PRIMARY_KEY - unique identifier of records in the patient database

FACZONE - a spatial aggregation of hospital facilities, which allows us to provide regional predictions of hospital bed need, not necessarily facility specific

SEX - the sex of the patient

UOA - unit of analysis is the spatial extent of aggregation for patient data (e.g., Zip Code, County) LOS - length of stay

AGEGRP - categorical assignment of age range for patients

FACILITY - unique code that identifies specific hospital facilities represented in the database

### Calculations

Here we present a step-by-step summary for the calculation of bed need projections. The specific implementation of the code for the 2011 Michigan Bed Need is available (see Additional File 1). The following SQL code assumes established tables for patient discharges (discharges), population statistics for each UOA (popStats) with variables for the base or current year population (popC) and projected count (popF), and targeted occupancy rates for hospital facilities in each ZONE.

Step 1: All hospital discharges for normal newborns (DRG code 391) and psychiatric patients (ICD9 codes 290 to 319) are excluded. To the remaining data, attach aggregation codes for hospitals (FACZONE). Index the resulting table on AGE, UOA, and FACILITY.

create table restricted

select * from discharges

where DRG < > '391' and ICD9 not between '290' and '319';

create table facility_zones

select restricted.PRIMARY_KEY, facilities.ZONE, restricted.SEX, restricted.UOA, facilities.UOA, restricted.LOS, restricted.AGEGRP, restricted.FACILITY from restricted INNER JOIN facilities on restricted.FACILITY = facilities.FACILITY;

create index AGEGRP on facility_zones (AGEGRP);

create index UOA on facility_zones (UOA);

create index FACILITY on facility_zones (FACILITY);

Step 2: For all discharges from each UOA, calculate the number of patient days (aggregated LOS) for each patient age group.

create table patient_days

select ZONE, UOA, AGEGRP, sum(LOS) as DAYS from facility_zones

group by ZONE, UOA, AGEGRP;

Step 3: Calculate a relevance index (Z) for each UOA to facility zones, which is essentially the ratio of UOA patient days to total patient days from each zone. This index defines the relative importance of the UOA in supplying patient days to that zone. The numerator includes patient days to hospitals of interest, whereas the denominator can include patient days to all hospitals from the UOA, regardless of whether they may be to hospitals outside of the state.

create table Znumerator

select ZONE, UOA, AGEGRP, DAYS as N from patient days;

create table Zdenominator

select UOA, AGEGRP, sum(DAYS) as D from patient_days

group by UOA, AGEGRP;

create table Zindex

select Znumerator.ZONE, Znumerator.UOA, Znumerator.AGEGRP,

Znumerator.N/Zdenominator.D as Z

from Znumerator INNER JOIN Zdenominator ON Znumerator.UOA = Zdenominator.UOA AND Znumerator.AGEGRP = Zdenominator.AGEGRP

group by ZONE, UOA, AGEGRP;

Step 4: For each ZONE, multiply the relevance index (Z) for UOA (Step 3) by its respective base year population (for each age group). The result is patient days by UOA and age group for each ZONE. Weight the populations from UOAs to ZONEs according to their specific Z value.

create table popJoin

select * from Zindex INNER JOIN popStats on Zindex.UOA = popStats.UOA

group by ZONE, UOA, AGEGRP;

create table weighted_pop

select ZONE, UOA, AGEGRP, Z * popC as weighted_pop from popJoin;

Step 5: For each ZONE, calculate the representative base year population for each age group by adding together the weighted UOA population allocations (Step 4). The result is base year population estimates for ZONEs by patient age groups.

create table ZonePopC

select ZONE, AGEGRP, sum(weighted_pop) as popC from weighted_pop

group by ZONE, AGEGRP;

Step 6: For each ZONE, calculate the hospital usage rates for a population for each age group by dividing the aggregated number of patient days for a ZONE (Step 2) by the weighted ZONE population (Step 5).

create table ZonePatientDays

select ZONE, AGEGRP, sum(DAYS) as DAYS from patient_days

group by ZONE, AGEGRP;

create table UseRates

select ZonePatientDays.ZONE, ZonePatientDays.AGEGRP, ZonePatientDays.DAYS/ZonePopC.popC as USERATE from ZonePatientDays INNER JOIN ZonePopC ON ZonePatientDays.ZONE = ZonePopC.ZONE AND ZonePatientDays.AGEGRP = ZonePopC.AGEGRP

group by ZONE, AGEGRP;

Step 7: For each ZONE, multiply each UOA's Z value (Step 3) by its respective projected population (popF) for each age group. The result is weighted population estimates for each UOA to ZONEs.

create table weighted_popF

select ZONE, UOA, AGEGRP, Z * popF as weighted_popF from popJoin;

Step 8: For each ZONE, calculate the total projected population (popF) by adding together each UOA allocation (Step 7).

create table ZonePopF

select ZONE, AGEGRP, sum(weighted_popF) as popF from weighted_popF

group by ZONE, AGEGRP;

Step 9: For each ZONE, calculate the projected patient days multiplying the projected populations by age group (Step 8) by the age specific use rates (Step 6).

create table patient_daysF

select ZonePopF.ZONE, ZonePopF.AGEGRP, ZonePopF.popF * UseRates.USERATE as daysF from ZonePopF INNER JOIN UseRates ON ZonePopF.ZONE = UseRates.ZONE AND ZonePopF.AGEGRP = UseRates.AGEGRP

group by ZONE, AGEGRP;

Step 10: For each ZONE, aggregate the patient days three categories: adult medical/surgical, pediatrics (Ages 0- 14), and obstetrics patients. CON specifications require reporting these totals separately.

create table daysMED

select ZONE, AGEGRP, sum(daysF) as daysF from patient_daysF

where AGEGRP <> '0 to 14' and AGEGRP <> 'OB'

group by ZONE;

alter table daysMED

set AGEGRP = 'MED'

create table daysPED

select ZONE, AGEGRP, sum(daysF) as daysF from patient_daysF

where AGEGRP = '0 to 14'

group by ZONE;

create table daysOB

select ZONE, AGEGRP, sum(daysF) as daysF from patient_daysF

where AGEGRP = 'OB'

group by ZONE;

create table daysF

select * from daysMED, daysPED, daysOB;

Step 11: For each ZONE, projected average daily census (ADC) for each AGEGRP (Step 10) by dividing the projected patient days (daysF) by 365 (or 366 for leap year estimates). Round ADC up to a whole number.

create table ADC

select ZONE, AGEGRP, ceiling(sum(daysF))/365 as ADC from daysF

group by ZONE, AGEGRP;

Step 12: For each ZONE, select the appropriate occupancy rate as defined in the current hospital standards.

create table occ_join

select ZONE, AGEGRP, ADC.ADC, occMED, occPED, occOB from ADC, occupancyrates

where ADC.ADC = occupancyrates.ADC;

Step 13: For each ZONE and AGEGRP, calculate the projected bed need by dividing the ADC (Step 11) by the occupancy rate (Step 12). Round any part of a bed up to a whole number.

create table bed_need

select * from occ_join;

alter table bed_need

add column beds FLOAT;

update bed_need

set beds = ceiling(ADC/occMED) where AGEGRP = 'MED';

update bed_need

set beds = ADC/occPED where AGEGRP = 'PED';

update bed_need

set beds = ADC/occOB where AGEGRP = 'OB';

### Challenges

Several problems manifested that were not discussed in existing regulations. Out of State (OoS) hospital visits fall outside the purview of the CON, but their activities affect the Bed Need Methodology in several ways. Michigan residents utilizing OoS hospitals were counted in the denominator of the relevance index for each zip code according to their specific age group. This has the effect of decreasing the relevance index for every hospital subarea with OoS visits. Subsequently, after calculating preliminary average daily census (ADC) estimates for the planning year (2011), these are added back in. That is, we estimate the number of daily beds per zip code heading out of state, and then allocate them to subareas based on the relevance index. OoS patients utilizing Michigan hospitals have no effect on calculating the relevance index or on the planning year (2011) projections, since they are not coming from Michigan zip codes. However, they have the effect of utilizing bed days, and must therefore be accounted for. Base year OoS patient days are added to each planning year subarea total for the projected ADC. Since no projections for future OoS patients exist, we use raw counts from the 2006 patient data in the MIDB.

Not surprisingly, there are occasionally errors in patient data entered into the MIDB. In several cases, we identified patient records with zip codes that could not be matched with any zip code record in the projected population. In such cases, the records were assigned to the zip code of the hospital where the patient received treatment.

## Results

Implications of the established Bed Need Methodology are explored by considering its generated results for Michigan hospitals for the 2011 planning year (see Additional File 2). Of the 63 subareas in Michigan, 52 experienced an increase in bed need from 2006, while 11 saw a decrease. Overall, the state experienced a 4.3 percent increase in total bed demand per day, from 20,168 to 21,039. However, this projected need is still less than the 2006 inventory of 26,878 beds, suggesting that occupancy rates will be far lower in the next few years than desired. Only three subareas currently have a lower inventory than the model predicts they will require in 2011, and these deficits are small (4-5 beds per day). Other subareas are predicted to have surpluses of dozens to hundreds of beds per day.

The impact of the contribution of OoS patients and OoS hospital visits to the overall bed need projections was minimal. Table 1 reports the average daily census (ADC) and itemizes OoS influence for each subarea. Although this influence is generally low, the ADC for several subareas is more profoundly impacted, especially in the Upper Peninsula of Michigan. This is not unexpected considering much of the Upper Peninsula is deemed a limited access area (greater than 30 min travel time to the nearest hospital; see Messina et. al [3] for details).

**Table 1:** Average Daily Census Contributions

**FSA**	**MI Residents to MI Hosp**	**Out State Residents to MI Hosp**	**MI Residents to Out State Hosp**	**Total**	**FSA**	**MI Residents to MI Hosp**	**Out State Residents to MI Hosp**	**MI Residents to Out State Hosp**	**Total**
	
1A	2387	2	34	2423	5A	46	0	1	47
1B	372	1	5	378	5B	902	1	14	917
1C	1179	1	21	1201	5C	70	1	1	72
					
1D	2411	5	38	2454	6A	58	1	2	61
1E	379	1	6	386	6B	35	0	1	36
1F	547	1	7	555	6C	22	0	1	23
1G	191	1	3	195	6D	120	1	4	125
1H	1274	9	49	1332	6E	240	1	4	245
1I	29	0	1	30	6F	618	1	13	632
1J	102	1	20	123	6G	26	1	1	28
					
2A	667	1	13	681	6H	8	0	1	9
2B	201	1	22	224	6I	13	0	1	14
					
2C	28	1	4	33	7A	21	1	1	23
2D	60	1	14	75	7B	137	1	3	141
					
3A	645	2	25	672	7C	11	0	1	12
3B	199	2	6	207	7D	19	1	1	21
3C	185	1	19	205	7E	64	0	2	66
3D	40	0	13	53	7F	283	1	6	290
3E	39	1	3	43	7G	35	0	1	36
					
4A	36	0	1	37	7H	34	1	1	36
4B	28	1	1	30	7I	19	0	1	20
					
4C	10	0	1	11	8A	10	2	5	17
4D	6	0	1	7	8B	6	1	1	8
4E	22	0	1	23	8C	11	1	2	14
4F	84	0	2	86	8D	6	0	1	7
4G	269	1	5	275	8E	28	1	3	32
4H	1085	1	16	1102	8F	39	9	10	58
4I	27	0	1	28	8G	140	1	15	156
4J	97	1	2	100	8H	26	1	4	31
4K	9	1	1	11	8I	3	1	1	5
4L	16	0	1	17	8J	4	1	1	6
					8K	5	0	1	6
					8L	29	1	1	31

### Sensitivity Analysis

We investigated the use of alternative sources of population information and varying spatial resolutions on the impact of the final bed need projections. The current implementation of the methodology utilizes Zip code-level Claritas population projections; here we contrast the resulting estimates with those obtained using population projections from other sources (GeoLytics and the US Census Bureau). To do this, we translate the population projections provided at zip codes to projections at the county level, and re-computed results using both methods of spatial aggregation.

To aggregate zip code populations to counties, we used area-weighted summations so that each county receives a proportion of the zip code population based on the proportion of the area of the zip code falling in the county. Therefore, each county population is the sum of all population proportions of each zip code within it. The process assumes population densities are equal across each zip code, which is certainly not true, a source of error in the aggregation estimates.

Table 2 presents the results of a comparison of population estimates at the county level for each of the three data sources for years 2002, 2005, 2007, and 2011. In general, Claritas and GeoLytics data are not very different from the US Census data based estimates. Differences ranged from -0.56% to 1.44%. However, there were substantial differences in the projections for individual counties. In Table 3, we present estimates for Chippewa, Ingham, Ionia, Jackson, Otsego, and Wayne counties. Though these tables present data from years not directly employed in the official bed need computation, they illustrate the impact of changing spatial resolution on population estimation.

**Table 2:** Statewide Population Projections utilizing a range of different sources. Both Claritas and GeoLytics populations are computed at the zip code-level. U.S. Census estimates are computed at the county level.

	2002	2005	2007	2011
**Claritas**	9,994,437 (est.)	N/A	10,217,151 (proj.)	10,355,401 (proj.)
**GeoLytics**	N/A	10,100,695	N/A	10,336,639
**U.S. Census**	10,050,446	10,207,421	10,071,822	N/A
**Percent Difference**	-0.56	-1.05	1.44	0.18

**Table 3:** The difference between the GeoLytics and Claritas population estimates from US Census estimates for 2005 and 2007. Claritas data varied to a greater degree than GeoLytics estimates. Aggregating across all counties to compute statewide totals dampened the overall difference from US Census estimates.

County	GeoLytics 2005 Projection	US Census 2005 Estimate	Percent Difference	Claritas 2007 Projection	US Census 2007 Estimate	Percent Difference
Chippewa	38,844	38,602	0.01	39,951	38,922	0.03
Ingham	278,119	281,002	-0.01	234,976	279,295	-0.16
Ionia	64,468	63,891	0.01	65,309	64,053	0.02
Jackson	163,432	162,702	0.00	168,173	163,006	0.03
Otsego	24,608	24,306	0.01	24,980	24,223	0.03
Wayne	1,990,932	2,027,238	-0.02	1,436,542	1,985,101	-0.28
						
Statewide	10,100,695	10,107,940	0.00	9,441,691	10,071,822	-0.06

To explore the impact of spatial resolution on bed need totals, we recomputed bed need using the population estimates from Claritas aggregated to county-level units. There were 130 more beds projected using counties rather than zip codes, less than a 1% difference. However, this discrepancy may be due to the overall higher population projected using the modified method (Table 1 vs. 2). GeoLytics data were also used to compute bed need at the zip code and county level; the total statewide difference was only 108 beds (0.5%).

Choice of population data source had a moderate impact on higher bed need projections at the zip code level; using GeoLytics instead of Claritas resulted in a 0.8% increase (180 beds). The majority of the bed differences were in the southeastern portion of the state. A county-level data comparison of GeoLytics versus Claritas also yielded higher projections using GeoLytics data (an increase of 158 beds or 0.7%). Finally, we calculated bed need totals using 2007 US Census population estimates with 2006 utilization rates. This total was lower than the Claritas projection by 293 beds, a decrease of 1.43%.

## Discussion

The current language in the hospital standards that details the Bed Need Methodology requires the use of a specific commercial vendor (Claritas) for supplying population projections for Michigan. This is in part a result of the requirement that bed need be calculated for zip codes instead of counties or other spatial units within the state. Claritas is one of only a few vendors to produce projections at such a fine spatial resolution. While we make no judgments as to the validity of their projections, we note that their methods tend to have predicted an approximately linear growth in population for Michigan between 2002 and 2011. Conversely, the Michigan Department for Census and Statistical Data (a division of the History, Arts, and Libraries (HAL)), estimates that Michigan is currently experiencing a decline in population. The nature of the methodology for Bed Need projections dictates that if a population growth is predicted, an increase in bed need will inevitably follow. Furthermore, there is no consideration for a potential change in the utilization rate of patients for hospitals; however due to the short prediction estimate (5 yrs) we would not anticipate utilization rates to change significantly. The use of a commercial vendor for population projections products casts uncertainty on the resulting product due to the proprietary nature of the vendor's product and lack of published methodology. This concern is supported by the apparent contradiction between Claritas' projections and the population estimates published by the State of Michigan.

Another complication of the current methodology is the dynamic nature of zip code designations across Michigan. Every year, zip codes split or merge in Michigan; however, the authors are not aware of any single repository for describing these changes, either spatially or in designation. It is therefore difficult to re-assign populations to new zip codes, as well as to predict how those populations will change in the future. Furthermore, since MIDB zip code designations are provided by the patient there is some uncertainty associated with the reliability of the information.

The Michigan implementation of the Bed Need Methodology is unique. Compared with the methods employed by the other states explored here with similar demographic and socio-economic populations (see Additional File 3), only Illinois, Iowa, and New York utilize a CON commission (i.e. states that project acute care bed need into the future for regulatory purposes). Further, most of these states calculate hospital bed need based on county level population statistics. The exceptions are Illinois and New York, which aggregate their county level projections to peer county groupings defined by region and assuming comparable demographic and socio-economic conditions.

In Illinois, the Health Facilities Planning Board created 40 planning areas comprised of counties, townships, and neighborhood regions. These planning areas are distributed throughout six regions that share boundaries with Health Service Areas (HSAs). The determination of beds is made through the calculation of age specific base year use rates using the average of three years of age specific patient days divided by a base year population for each age group. Projected patient days are calculated by multiplying the age specific use rate by a ten-year population projection by age group and migration factor [4].

Similarly, New York, with eight peer county groups, utilizes a base year population estimation and projects bed need five years into the future (as does Michigan). Bed need is determined using a normalized hospital discharge rate per 1,000 population by age and gender. The discharge rate excludes neonatal discharges, newborns, and discharges with non-medical/surgical DRG codes (maternity, psychiatric, drug abuse, alcohol abuse, burns, medical rehabilitation, and HIV patients). The discharge rates are calculated for each county and used to project bed need by multiply by the county level population projections. The population projections are computed by age and gender in- house by the NYS Department of Economic Development [5].

Iowa calculates bed need based on a computation of statewide patient days. An annually adjusted patient day is calculated for each age group and county designation. These adjusted days are used to calculate patient-day use rates by age group, which are then projected ten years out, utilizing county level population projections. As was the case in New York, Iowa computes population projections in- house and published by the Iowa Department of Economic Development [6].

While Indiana and Ohio do not have CON programs for acute care facilities, they do employ CON commissions for the management of long-term care facilities. The method for projecting bed need in both states is based on 4-year projections at the county level. These states use population projections computed in-house by the Indiana Business Research Center and the Ohio Department of Development, respectively [7,8]. Although the projection of bed need in these cases is for long-term care facilities, the general methodologies are still comparable to the methods presented in this paper.

In a review of international methods of projecting subacute care demand, Gibbs et al. [9] illustrated the many ways in which hospitals and oversight agencies have calculated bed need projections. While Gibbs et al. focused on subacute care, the methods for subacute care were often in relation to acute care projection models. As there is a decreasing trend in the acute care length of stay some of these methods suggest adding a trend variable to account for the national tendency of declining acute care bed need. According to Steven Sauer, a consultant with Hamilton/KSA in Minneapolis, inpatient utilization rates in 1980 in the USA were nationally 1,217 days per thousand population for commercial patients, and this rate fell to 795 days in 1991 [10]. This suggests the integration of a variable to account for the national trend of declining bed need into future projections may yield more accurate bed need projections. Another method is to apply a strict benchmark or ratio of beds per 100,000 population, as is currently undertaken in Ontario, Canada [10]. The general benchmark in the United States for utilization rates is currently 248 days per thousand population and he suggests this number could be as low as 180 days per thousand population [10]. However, the most frequent method of bed need projection researched by Gibbs et al. was the employment of utilization rates, either current or trended, applied to a projected population. These included utilization rates at various levels of aggregation and different demographics.

A study of bed need in Greece that attempted to project need by sex and age groups utilizes ten age groups, rather than the five the MDCH relies upon [11]. This methodology calculates the minimum and maximum number of male and female admissions for each year and population projections based on a logistic function using data over the past ten years. The number of projected admissions is calculated though the summation of the totals of the minimum and maximum values by each age group and sex. To calculate the number of hospital beds required, Mouza [11] simply multiplies the number of admissions by the mean length of stay and divides the result by the result of 365 multiplied by the occupancy rate. Mouza also introduces a time-trend variable to the weighted mean length of stay to weight the most recent values more heavily, rather than weighing all values equally. Thus, multiplying the weighted mean length of stay by the minimum and maximum values of the number of projected admissions, dividing the result by the result of 365, and multiplying by the occupancy rate, provides an upper and lower bound to the number of beds that will be required, rather than a single value.

Researchers from the Manitoba Centre for Health Policy at the University of Manitoba used the Trends in Acute Care Bed Use model to predict the number of acute care hospital beds in the province of Manitoba for the year 2020 [12]. This model utilizes a ten-year history of bed use for experience with a stratified regression model to predict trends and rates of change, rather than current use and projections of that use into the future under the Current Use Projection model.

Aside from calculating bed need, researchers in New South Wales, Australia suggest the projection of acute patient activity rather than a projection of bed numbers. Jones et al. [13] use the alM2005 model to project the amount of acute patient activity, and ultimately, the volume and type of work into the future. The alM2005 model utilizes admitted patient data, small-area population estimates, and population projections of statistical local areas. The model employs a regression analysis to predict trends of enhanced related service groups, age, sex, and length of stay into the future using bounded growth rates to account for early data points that may not reflect the underlying trend within the data. While the input variables are similar to those used in our paper, the methodologies of the production of future use rates differ.

To summarize, the methodologies for the states compared here show that bed need assessments are done at a number of varying spatial scales; however, Michigan is the only state to perform such analysis at the zip code level. There is no convention as to how many years into the future bed need projections are calculated. Intervals range from 3 to 10 years. Majorities of these states perform population projections in-house; Michigan is the only state that purchases projections from a private vendor. In light of these comparisons, it seems reasonable to suggest MDCH re-evaluate their selected unit of analysis. This could facilitate a move to calculate population projections in-house.

## Summary

This paper provides a detailed description of the calculation of Bed Need projections for the State of Michigan as described by the Hospital Standards and mandated by the Michigan State Legislature. The methods presented here represent our best interpretation of the intent of the methodology as laid out by the Michigan CON. The method is presented using generalized SQL code to facilitate broad application of the methodology. Our methodology utilizes patient records collected by the Michigan Health Association and made available to the authors by the Michigan Department of Community Health. In short, the methodology calculates a use rate for patient zip codes to each hospital subarea in the base year and multiplies this rate by the projected population within each age group and zip code across the state to obtain a total projected bed need by subarea. The resulting bed need is adjusted according to the desired occupancy rate, and then divided into three distinct groups: adult medical/surgical, obstetrics, and pediatrics.

The methodology for the State of Michigan is different from the methodologies of other states, primarily in the use of zip code as the unit of analysis. This necessitates the use of a commercial vendor for population projections, the only state reported to do so. Other states examined in this study have utilized in-house county level (or larger) population projection data, which may result in projections that are more accurate since errors are amplified with small zip codes and small population sizes. Exacerbating factors include the impact of migration, growth, and utilization rates, as well as measurement error, on small populations. Furthermore, utilizing a commercial vendor for population projections where the methodology is restricted increases output uncertainty, especially considering the declining real population across the state and the projected increase in population in the provided population projection data.

Altering the data source and the spatial unit of analysis was found to have a modest impact on the outcome of bed need projections statewide. However, larger differences were observed at the zip code or county level. Therefore, although bed need is computed at an intermediate level between zip code and statewide, the selection of a spatial unit can significantly affect the computation of bed need for a region. However, the historic over-estimates of bed need are not solely the result of inaccurate population projections. This leads us to believe that some aspect of the computation of bed need unintentionally inflates the need. Though we do not test for this explicitly, incorporating a distribution of utilization rates may capture the uncertainty in the projections.

This paper addresses the need by State and Hospital officials for a clear explanation of the steps required to compute the bed need methodology as presented in the current Hospital Standards. The method is presented in sufficient detail (with corresponding SQL code included in appendix) such that an interested party could replicate our results with the appropriate data sets, compare these results with those from an alternative approach, or implement this bed need methodology in another region, for other times.

## Competing interests

The authors declare that they have no competing interests.

## Authors' contributions

SAL developed the code for the methodology presented here, and drafted the manuscript. SPF assisted in verifying the SQL code and drafting the manuscript as it pertains to neighboring states' utilization of bed need methodologies. JPM and AMS assisted in the development of the SQL code, development of the manuscript, and in the dissemination of the results to the Michigan Department of Community Health. SCG assisted in the review of the methods and population models. All authors have read and approved the final version of this manuscript.

## Supplementary Material

Additional file 1 **SQL code for calculating bed need values for planning year 2011**. Our specific implementation of the bed need methodology for planning year 2011 is generated using the SQL code given here. Click here for file

Additional file 2 **Results of the bed need computation for planning year 2011 **The results of our computation of bed need for planning year 2011, utilizing the code given in Additional File 1, are presented here. We compare the bed need for 2011 with the previously reported bed need for planning year 2006, as well as the hospital bed inventory in 2006. The resulting unmet bed need (or excess) is reported. Click here for file

Additional file 3 **A comparison of bed need computation by state**. A comparison of bed need methodologies as implemented by a select grouping of states. Click here for file
