# Supplementary material for: A methodology for projecting hospital bed need: a Michigan case study
Source: Source Code Biol Med. 2010 Mar 25;5:4. doi: 10.1186/1751-0473-5-4 (PMC2861647; doi:10.1186/1751-0473-5-4)
Supplement: Additional file 1 — SQL code for calculating bed need values for planning year 2011. Our specific implementation of the bed need methodology for planning year 2011 is generated using the SQL code given here. [file 1751-0473-5-4-S1.PDF]

Additional file 1

SQL Code for Calculating Bed Need Values for Planning Year 2011.

*/*Parent tables: discharge06, claritas08, hosp_key08, occupancy*
Note: these tables are written permanently into the database/*

/*Step 1: All hospital discharges for normal newborns (DRG 391) and psychiatric patients (ICD9CM 290 to 319) are excluded.*/

```
drop table restrict1b;
create table restrict1b
select * from discharge06
where HCFA_DRG <> '391' AND PRINC_DX not between '290' and '319' AND AGE <> '199';

create index AGE on restrict1b (AGE);
create index ZIP on restrict1b (MIDB_ZIP);
create index HOSPID on restrict1b (HOSP_ID);
create index MKEY on restrict1b (MKEY);
```

/*Add age group divisions.*/

```
alter table restrict1b
add age_grp varchar(12);

update restrict1b
set age_grp='0 to 14' where AGE between '0' and '14';
update restrict1b
set age_grp='15 to 44' where AGE between '15' and '44' and HCFA_DRG not between '370' and '375';
update restrict1b
set age_grp='OB' where AGE between '15' and '44' and HCFA_DRG between '370' and '375';
update restrict1b
set age_grp='45 to 64' where AGE between '45' and '64';
update restrict1b
set age_grp='65 to 74' where AGE between '65' and '74';
update restrict1b
set age_grp='75+' where AGE >= '75';
update restrict1b
set age_grp='0 to 14' where AGE='190';
update restrict1b
set age_grp='0 to 14' where AGE='195';
```

/*Attach subarea data for hospitals.*/

```
drop table hospmerge;
create table hospmerge
SELECT
restrict1b.MKEY,
hosp_key08.hsa,
hosp_key08.fsa,
restrict1b.SEX,
restrict1b.ZIP,
restrict1b.MIDB_ZIP,
hosp_key08.hosp_zip,
restrict1b.LOS,
restrict1b.age_grp,
restrict1b.HOSP_ID
FROM
restrict1b
INNER Join hosp_key08 ON restrict1b.HOSP_ID = hosp_key08.hosp_id;

create index MKEY on hospmerge (MKEY);
create index ZIP on hospmerge (MIDB_ZIP);
create index HospID on hospmerge (HOSP_ID);

/* For those zipcodes that are not in the claritas08 dataset (see table badzip) I will replace the
zipcode with the zipcode of the hospital identified in the discharge record */

update hospmerge
set MIDB_ZIP = ZIP where MIDB_ZIP in (select MIDB_ZIP from badzip);

update hospmerge
set MIDB_ZIP = hosp_zip where MIDB_ZIP in (select MIDB_ZIP from badzip);


/*Step 2: For each discharge from the selected zip, calculate the number of patient days for each
age group.  Data from non-Michigan residents are to be included for each specific age group (i.e.
all zip codes, fsa = null designate out of state hospitals).*/

drop table patient_days;
create table patient_days
select fsa, MIDB_ZIP, age_grp, sum(LOS) AS DAYS
from hospmerge
where MIDB_ZIP is not null
group by fsa, MIDB_ZIP, age_grp;

/*Step 3: For each FSA, calculate the relevance index (%Z) for each zip and for each age group.
Numerators should include all patient days to a Michigan hospital; whereas denominators should
include all patients */
```

/* Patient days for out of state hospitals are separated with fsa=9 and therefore don't affect the calculations for instate hospitals. However it will allow other calculations to made later. */

```
drop table Znumerator;
create table Znumerator
select fsa, MIDB_ZIP, age_grp, DAYS as ZNUM
from patient_days
group by fsa, MIDB_ZIP, age_grp;

create index ZIP on Znumerator (MIDB_ZIP);

alter table Znumerator
modify column ZNUM FLOAT(12);

drop table Zdenominator;
create table Zdenominator
select MIDB_ZIP, age_grp, sum(DAYS) as ZDENOM
from patient_days
group by MIDB_ZIP, age_grp;

create index ZIP on Zdenominator (MIDB_ZIP);

alter table Zdenominator
modify column ZDENOM FLOAT(12);

drop table Zmerge;
create table Zmerge
select Znumerator.fsa,
Znumerator.MIDB_ZIP,
Znumerator.age_grp,
Znumerator.ZNUM,
Zdenominator.ZDENOM
from Znumerator
INNER Join Zdenominator ON Znumerator.MIDB_ZIP = Zdenominator.MIDB_ZIP AND
Znumerator.age_grp = Zdenominator.age_grp
group by fsa, MIDB_ZIP, age_grp;

drop table Zindex;
create table Zindex
select fsa, MIDB_ZIP, age_grp, ZNUM/ZDENOM as Zvalue
from Zmerge;

alter table Zindex
modify column Zvalue FLOAT(12);
```

/*Step 4: For each FSA, multiply each zip %Z calculated in step 3 by its respective base year zip code and age group specific year population. The result will be the zip code allocations by age group for each FSA.*/

/* Join claritas population data to Zvalue table.*/

```
drop table popjoina;
Create Table popjoina
Select * from Zindex INNER JOIN claritas08 ON Zindex.MIDB_ZIP = claritas08.ZIPCODE
group by fsa, MIDB_ZIP, age_grp;

create index AGE on popjoina (age_grp);

alter table popjoina
add column popC FLOAT(12);

update popjoina
set popC = (POPM_0_4_C + POPM_5_9_C + POPM10_14C + POPF_0_4_C + POPF_5_9_C +
POPF10_14C) where age_grp = '0 to 14';

update popjoina
set popC = (POPM15_17C + POPM18_20C + POPM21_24C + POPM25_34C + POPM35_44C +
POPF15_17C + POPF18_20C + POPF21_24C + POPF25_34C + POPF35_44C) where age_grp = '15
to 44';

update popjoina
set popC = (POPF15_17C + POPF18_20C + POPF21_24C + POPF25_34C + POPF35_44C) where
age_grp = 'OB';

update popjoina
set popC = (POPM45_49C + POPM50_54C + POPM55_59C + POPM60_64C + POPF45_49C +
POPF50_54C + POPF55_59C + POPF60_64C) where age_grp = '45 to 64';

update popjoina
set popC = (POPM65_74C + POPF65_74C) where age_grp = '65 to 74';

update popjoina
set popC = (POPM75_84C + POPM_85P_C + POPF75_84C + POPF_85P_C) where age_grp = '75+';

alter table popjoina
add column popF FLOAT(12);

update popjoina
set popF = (POPM_0_4_F + POPM_5_9_F + POPM10_14F + POPF_0_4_F + POPF_5_9_F +
POPF10_14F) where age_grp = '0 to 14';

update popjoina
```

```
set popF = (POPM15_17F + POPM18_20F + POPM21_24F + POPM25_34F + POPM35_44F +
POPF15_17F + POPF18_20F + POPF21_24F + POPF25_34F + POPF35_44F) where age_grp = '15 to
44';

update popjoina
set popF = (POPF15_17F + POPF18_20F + POPF21_24F + POPF25_34F + POPF35_44F) where
age_grp = 'OB';

update popjoina
set popF = (POPM45_49F + POPM50_54F + POPM55_59F + POPM60_64F + POPF45_49F +
POPF50_54F + POPF55_59F + POPF60_64F) where age_grp = '45 to 64';

update popjoina
set popF = (POPM65_74F + POPF65_74F) where age_grp = '65 to 74';

update popjoina
set popF = (POPM75_84F + POPM_85P_F + POPF75_84F + POPF_85P_F) where age_grp = '75+';

drop table popjoinb;
create  table popjoinb
select fsa, MIDB_ZIP, age_grp, Zvalue, popC, popF from popjoina;

/* Here we produce weighted populations for Mich Zips to all hospitals */

drop table Zallocate_baseyr;
create table Zallocate_baseyr
select fsa, MIDB_ZIP, age_grp, Zvalue * popC AS Zip_allocation
from popjoinb
where fsa <> '9';

/*Step 5: For each FSA, calculate the subarea base year population by age group by adding
together all zip code population allocations calcualted in step 4 for each specific age group in that
FSA.  The result will be six population age groups for each FSA.*/

drop table FSApop_baseyr;
create table FSApop_baseyr
select fsa, age_grp, sum(Zip_allocation) AS POP
from Zallocate_baseyr
group by fsa, age_grp;

create index fsa on FSApop_baseyr (fsa);
create index age_grp on FSApop_baseyr (age_grp);

/*Step 6: For each FSA, calculate the patient day use rates for ages 0 to 14, 15-44, OB, 45-64, 65-74, and 75+ by
dividing the results of step 2 by the results of step 5.*/
```

```
drop table patient_days_consolidate;
create table patient_days_consolidate
select fsa, age_grp, sum(DAYS) as DAYS
from patient_days
group by fsa, age_grp;

create index fsa on patient_days_consolidate (fsa);
create index age_grp on patient_days_consolidate (age_grp);


drop table patient_userates;
create table patient_userates
select patient_days_consolidate.fsa, patient_days_consolidate.age_grp,
patient_days_consolidate.DAYS / FSApop_baseyr.POP AS Rate
from patient_days_consolidate INNER JOIN FSApop_baseyr ON patient_days_consolidate.fsa =
FSApop_baseyr.fsa AND patient_days_consolidate.age_grp = FSApop_baseyr.age_grp
group by fsa, age_grp;

/*Step 7: For each FSA, multiply each zip code %Z calculated in step 3 by its respective planning
year zip code and age group specific year population.  The results will be the projected zip code
allocations by age group for each FSA.*/

drop table Zallocate_planyr;
create table Zallocate_planyr
select fsa, MIDB_ZIP, age_grp, Zvalue * popF AS Zip_allocation
from popjoinb
where fsa <> '9';

/* Here I'm estimating the population for Mich hospitals where Mich. residents attended */

create index fsa on Zallocate_planyr (fsa);
create index age_grp on Zallocate_planyr (age_grp);


/*Step 8: For each FSA, calculate the subarea projected year population by age group by adding
together all projected zip code population allocations calculated in step 7 for each specific age
group.  The result will be six population age groups for each FSA.*/

drop table FSApop_planyr;
create table FSApop_planyr
select fsa, age_grp, sum(Zip_allocation) AS POP
from Zallocate_planyr
group by fsa, age_grp;

create index fsa on FSApop_planyr (fsa);
```

```
create index age_grp on FSApop_planyr (age_grp);
```

/*Step 9: For each FSA, calculate the FSA projected patient days for each age group by multiplying six projected populations by age group calculated in step 8 by the age specific use rates identified in step 6.*/

```
drop table patient_days_projected_1;
create table patient_days_projected_1
select FSApop_planyr.fsa, FSApop_planyr.age_grp, FSApop_planyr.POP * patient_userates.Rate as
DAYS_proj
from FSApop_planyr INNER JOIN patient_userates ON FSApop_planyr.fsa = patient_userates.fsa
AND FSApop_planyr.age_grp = patient_userates.age_grp
group by fsa, age_grp;
```

/* Calculate the patient days contribution from mich residents to out of state hospitlas */

/* A weighted population projection for zipcodes where patient went out of state. We will use these new weighted population to re-distribute to instate hospitals */

```
drop table outstate_pop;
create table outstate_pop
select MIDB_ZIP, age_grp, Zvalue * popF AS POP
from popjoinb
where fsa = '9';
```

/* Now we have a new Zip code population that we can pretend is the total projected population for each zip and recalculate projected patient days */

```
drop table outstate_popjoin;
create table outstate_popjoin
select Zindex.fsa, outstate_pop.MIDB_ZIP, outstate_pop.age_grp, outstate_pop.POP, Zindex.Zvalue
from Zindex INNER JOIN outstate_pop on Zindex.MIDB_ZIP = outstate_pop.MIDB_ZIP and
Zindex.age_grp = outstate_pop.age_grp;

drop table outstate_Zallocate_planyr;
create table outstate_Zallocate_planyr
select fsa, MIDB_ZIP, age_grp, Zvalue * POP AS Zip_allocation
from outstate_popjoin
where fsa <> '9';

create index fsa on outstate_Zallocate_planyr (fsa);
create index age_grp on outstate_Zallocate_planyr (age_grp);

drop table outstate_FSApop_planyr;
create table outstate_FSApop_planyr
select fsa, age_grp, sum(Zip_allocation) AS POP
```

```
from outstate_Zallocate_planyr
group by fsa, age_grp;

create index fsa on outstate_FSApop_planyr (fsa);
create index age_grp on outstate_FSApop_planyr (age_grp);

drop table outstate_patient_days_projected;
create table outstate_patient_days_projected
select outstate_FSApop_planyr.fsa, outstate_FSApop_planyr.age_grp, outstate_FSApop_planyr.POP *
patient_userates.Rate as DAYS_proj
from outstate_FSApop_planyr INNER JOIN patient_userates ON outstate_FSApop_planyr.fsa =
patient_userates.fsa AND outstate_FSApop_planyr.age_grp = patient_userates.age_grp
group by fsa, age_grp;

/* Calculate the raw patient days for out of state residents that attend a Mich Hospital */

drop table patient_days_outstate;
create table patient_days_outstate
select fsa, age_grp, sum(LOS) as DAYS
from hospmerge
where MIDB_ZIP in (select MIDB_ZIP from NonMich_Zips) AND fsa <> '9'
group by fsa, age_grp;


/* Pull out just the Michigan patient_days before altering the previous table so I can have this
information later to include in the report */

drop table mich_patient_days_projected;
create table mich_patient_days_projected
select * from patient_days_projected_1;

/* Add these raw patient days back into the full table of projected days */

insert into patient_days_projected_1 (fsa, age_grp, DAYS_proj)
select fsa, age_grp, DAYS from patient_days_outstate;


/* Add the patient days for instate patients that went out of state, but re-allocated to instate hospitals */

insert into patient_days_projected_1 (fsa, age_grp, DAYS_proj)
select fsa, age_grp, DAYS_proj from outstate_patient_days_projected;

/* So now we have patient days for Mich residents to Mich hospitals, Outstate residents to Mich
hospitals, and a reallocation of Mich Residents, who went out of state, to michigan hospitals */

drop table patient_days_projected;
```

```
create table patient_days_projected
select fsa, age_grp, sum(DAYS_proj) as DAYS_proj from patient_days_projected_1
group by fsa, age_grp;

/*separate patient days are listed as mich_patient_days_projected, patient_days_outstate, and
outstate_patient_days_projected.  We can calculate the ADC for those days next */

drop table ADCmich;
create table ADCmich
select fsa, ceiling(sum(DAYS_proj) / 365) as ADCmich
from mich_patient_days_projected
group by fsa;

drop table ADCoutstate;
create table ADCoutstate
select fsa, ceiling(sum(DAYS) / 365) as ADCoutstate
from patient_days_outstate
group by fsa;

drop table ADCreallocated;
create table ADCreallocated
select fsa, ceiling(sum(DAYS_proj) / 365) as ADCreallocate
from outstate_patient_days_projected
group by fsa;


/*Step 10: For each FSA, calculate the adult medical/surgical FSA projected patient days by
adding together the following age group specific projected patient days calculated in step 9: 15-
44, 45-64, 65-74, 75+.  The 0-14, and OB age groups remain unchanged as calculated in step 9.*/

drop table medical_days_1;
create table medical_days_1
select fsa, sum(DAYS_proj) as DAYS_proj
from patient_days_projected
where age_grp <> '0 to 14' AND age_grp <> 'OB'
group by fsa;

alter table medical_days_1
add column age_grp VARCHAR(12);

update medical_days_1
set age_grp = 'Medical';

drop table medical_days_2;
create  table medical_days_2
select *
```

```
from patient_days_projected
where age_grp = '0 to 14' or age_grp = 'OB';

insert into medical_days_1 (fsa, age_grp, DAYS_proj)
select fsa, age_grp, DAYS_proj from medical_days_2;

drop table medical_days;
create  table medical_days
select fsa, age_grp, DAYS_proj
from medical_days_1
order by fsa, age_grp;

/*Step 11: For each FSA, calculate the subarea projected average daily census (ADC) for three age
groups: 0-14, OB, and adult medical/surgical by dividing the results calculated in step 10 by 365
(or 366 for planning leap year).  Round each ADC to a whole number.  This will give three ADC
computations per FSA.*/

drop table ADC;
create  table ADC
select fsa, age_grp, ceiling(DAYS_proj / 365) AS ADC
from medical_days;


/*Step 12: For each FSA, select the appropriate occupancy rate from the occupancy rate table.*/

drop table occup_join;
create table occup_join
select fsa, age_grp, ADC.ADC, occ_rate_surgical, occ_rate_peds, occ_rate_ob
from ADC, occupancy
where ADC.ADC = occupancy.ADC;


/*Step 13: For each FSA and age group, calculate the FSA projected bed need number of hospital
beds for the FSA by age group by dividing the ADC calculated in step 11 by the appropriate
occupancy rate determined in step 12.  Round any part of a bed up to a whole bed.*/

drop table bed_need_1;
create table bed_need_1
select * from occup_join;

alter table bed_need_1
add column beds FLOAT;

update bed_need_1
set beds = ADC / occ_rate_surgical  where age_grp = 'Medical';
```

```
update bed_need_1
set beds = ADC / occ_rate_peds where age_grp = '0 to 14';

update bed_need_1
set beds = ADC / occ_rate_ob where age_grp = 'OB';

drop table bed_need;
create  table bed_need
select fsa, age_grp, ceiling(beds) as BEDS
from bed_need_1
group by fsa, age_grp;
```
