# Supplementary material for: A methodology for projecting hospital bed need: a Michigan case study
Source: Source Code Biol Med. 2010 Mar 25;5:4. doi: 10.1186/1751-0473-5-4 (PMC2861647; doi:10.1186/1751-0473-5-4)
Supplement: Additional file 2 — Results of the bed need computation for planning year 2011 The results of our computation of bed need for planning year 2011, utilizing the code given in Additional File 1, are presented here. We compare the bed need for 2011 with the previously reported bed need for planning year 2006, as well as the hospital bed inventory in 2006. The resulting unmet bed need (or excess) is reported. [file 1751-0473-5-4-S2.PDF]

Additional File 2

Results of the Bed Need Computation for Planning Year 2011

FSA	0 to 14	Medical	OB	Current Bed Need *	**Projected Bed Need ****	± Diff	% Change	**Projected Bed Need ****	2006 Inventory	Unmet Bed Need (Excess)
1A	210	2537	199	2732	**2946**	214	7.83	**2946**	3954	(1008)
1B	6	462	12	465	**480**	15	3.23	**480**	551	(71)
1C	75	1309	97	1497	**1481**	(16)	(1.07)	**1481**	2153	(672)
1D	366	2440	173	2966	**2979**	13	0.44	**2979**	4045	(1066)
1E	10	459	26	452	**495**	43	9.51	**495**	578	(83)
1F	30	636	34	673	**700**	27	4.01	**700**	770	(70)
1G	12	239	16	257	**267**	10	3.89	**267**	282	(15)
1H	227	1317	104	1571	**1648**	77	4.90	**1648**	1791	(143)
1I	2	45	6	50	**53**	3	6.00	**53**	68	(15)
1J	6	157	14	150	**177**	27	18.00	**177**	217	(40)
2A	104	704	81	841	**889**	48	5.71	**889**	1028	(139)
2B	18	264	24	375	**306**	(69)	(18.40)	**306**	389	(83)
2C	4	49	6	50	**59**	9	18.00	**59**	65	(6)
2D	8	95	14	90	**117**	27	30.00	**117**	113	4
3A	120	687	83	853	**890**	37	4.34	**890**	1116	(226)
3B	8	253	20	270	**281**	11	4.07	**281**	337	(56)
3C	14	248	20	233	**282**	49	21.03	**282**	419	(137)
3D	6	69	14	67	**89**	22	32.84	**89**	114	(25)
3E	4	61	6	61	**71**	10	16.39	**71**	102	(31)
4A	2	55	8	59	**65**	6	10.17	**65**	81	(16)
4B	4	40	8	51	**52**	1	1.96	**52**	99	(47)
4C	2	17		19	**19**	0	0.00	**19**	25	(6)
4D	2	9	2	13	**13**	0	0.00	**13**	24	(11)
4E	2	30	6	38	**38**	0	0.00	**38**	61	(23)
4F	8	111	14	145	**133**	(12)	(8.28)	**133**	191	(58)
4G	20	317	36	376	**373**	(3)	(0.80)	**373**	538	(165)
4H	220	1030	150	1340	**1400**	60	4.48	**1400**	1725	(325)
4I	2	42	4	42	**48**	6	14.29	**48**	87	(39)
4J	12	115	30	147	**157**	10	6.80	**157**	250	(93)
4K	2	14	2	18	**18**	0	0.00	**18**	25	(7)
4L	2	24	4	24	**30**	6	25.00	**30**	25	5
5A	4	66	8	81	**78**	(3)	(3.70)	**78**	111	(33)
5B	119	961	83	1126	**1163**	37	3.29	**1163**	1226	(63)
5C	2	99	8	117	**109**	(8)	(6.84)	**109**	183	(74)
6A	4	86	6	93	**96**	3	3.23	**96**	137	(41)
6B	6	50	6	56	**62**	6	10.71	**62**	118	(56)
6C	4	34	4	50	**42**	(8)	(16.00)	**42**	64	(22)
6D	8	159	14	174	**181**	7	4.02	**181**	255	(74)
6E	8	303	10	285	**321**	36	12.63	**321**	432	(111)
6F	85	687	48	764	**820**	56	7.33	**820**	1038	(218)
6G	2	40	6	38	**48**	10	26.32	**48**	106	(58)
6H	2	12	2	14	**16**	2	14.29	**16**	40	(24)
6I	2	20		26	**22**	(4)	(15.38)	**22**	35	(13)
7A	2	32	4	38	**38**	0	0.00	**38**	46	(8)
7B	10	176	14	188	**200**	12	6.38	**200**	254	(54)
7C		19		24	**19**	(5)	(20.83)	**19**	36	(17)
7D	2	29	4	32	**35**	3	9.38	**35**	46	(11)
7E	4	92	6	84	**102**	18	21.43	**102**	124	(22)
7F	26	340	26	374	**392**	18	4.81	**392**	393	(1)
7G	4	52	8	63	**64**	1	1.59	**64**	97	(33)
7H	2	53	4	57	**59**	2	3.51	**59**	90	(31)
7I	2	30	4	36	**36**	0	0.00	**36**	45	(9)
8A	2	24	4	21	**30**	9	42.86	**30**	25	5
8B	2	10		7	**12**	5	71.43	**12**	25	(13)
8C	2	20		19	**22**	3	15.79	**22**	25	(3)
8D	2	10		9	**12**	3	33.33	**12**	24	(12)
8E	4	44	6	54	**54**	0	0.00	**54**	61	(7)
8F	4	79	10	71	**93**	22	30.99	**93**	96	(3)

8G	30	182	14	211	**226**	15	7.11	**226**	289	(63)
8H	4	45	4	59	**53**	(6)	(10.17)	**53**	98	(45)
8I	2	5		6	**7**	1	16.67	**7**	11	(4)
8J	2	7		7	**9**	2	28.57	**9**	18	(9)
8K	2	9		7	**11**	4	57.14	**11**	25	(14)
8L	2	45	4	52	**51**	(1)	(1.92)	**51**	82	(31)
Totals	1864	17655	1520	20168	**21039**	871	4.32	**21039**	26878	(5839)

Bed Need Values for Planning Year 2011. The results of our implementation of the methods are shown in bold.

* Current Bed Need using base year 2004 data, planning year 2009

** Projected Bed Need using base year 2006 data, planning year 2011
