# Supplementary material for: A methodology for projecting hospital bed need: a Michigan case study
Source: Source Code Biol Med. 2010 Mar 25;5:4. doi: 10.1186/1751-0473-5-4 (PMC2861647; doi:10.1186/1751-0473-5-4)
Supplement: Additional file 3 — A comparison of bed need computation by state. A comparison of bed need methodologies as implemented by a select grouping of states. [file 1751-0473-5-4-S3.PDF]

Additional File 3

A Comparison of Bed Need Computation By State

State	Planning Interval	Unit of Analysis	Data Needed	Excluded Variables	Method of Projection	Population Data Source	C.O.N. State
Michigan	Base + 5 Years	Zip Code	Base year, population estimates	Newborns, Psych	Proprietary Model	Commercial	Yes (DCH)
Illinois	Base + 10 Years	40 Planning Areas, 6 Regions	Base year, previous 2 years patient-days by age group	N/A	Linear model from population estimates 10 years from base year	Statewide population projections	Yes (DPH)
Indiana	N/A	Hospital Owners	Market Forces	N/A	N/A	N/A	No
Iowa	Base (most recent survey) + 10 Years	County	Base year, patient-days by age group	N/A	Linear model from population estimates 10 years from base year	State Census & Iowa Dept. of Economic Development	Yes (DPH)
New York	Base + 5 Years	County, Peer Groupings	Base year, previous 5 years pop. by age group and sex	Newborns, Psych, burns, medical rehab, HIV/AIDS	Linear model from County projections	US Census & NYS Dept of Economic Development	Yes (DoH)
